# Streamlining staging of lung and colorectal cancer with whole body MRI; study protocols for two multicentre, non-randomised, single-arm, prospective diagnostic accuracy studies (Streamline C and Streamline L)

**DOI:** 10.1186/s12885-017-3281-x

**Published:** 2017-05-02

**Authors:** Stuart A. Taylor, Sue Mallett, Anne Miles, Sandy Beare, Gauraang Bhatnagar, John Bridgewater, Rob Glynne-Jones, Vicky Goh, Ashley M. Groves, Sam M. Janes, Dow Mu Koh, Steve Morris, Alison Morton, Neal Navani, Alf Oliver, Anwar R. Padhani, Shonit Punwani, Andrea G. Rockall, Steve Halligan

**Affiliations:** 1 Centre for Medical Imaging, University College London, 250 Euston Road, London, NW1 2BU UK; 2 School of Health and Population Sciences, University of Birmingham, Edgbaston, Birmingham, B15 2TT UK; 3 Birkbeck, University of London, Malet Street, London, WC1E 7HX UK; 4 Cancer Research UK & UCL Cancer Trials Centre, 90 Tottenham Court Road, London, W1T 4TJ UK; 5 UCL Cancer Institute, Paul O Gorman Building, 72 Huntley Street London, London, WC1E 6DD UK; 6 Mount Vernon Centre for Cancer Treatment, Rickmansworth Road, Northwood, MIDDX, HA6 2RN UK; 7 Cancer Imaging, Division of Imaging Sciences & Biomedical Engineering Kings College London, St Thomas Hospital, Westminster Bridge Road, London, SE1 7EH UK; 8 Institute of Nuclear Medicine, University College London, Euston Rd, London, UK; 9 Lungs for Living Research Centre, UCL Respiratory, University College London, 5 University Street, London, WC1E 6JF UK; 10 Department of Radiology, Royal Marsden Hospital, Downs Road, Sutton, SM2 5PT UK; 11 Department of Applied Health Research, University College London, 1-19 Torrington Pl, Fitzrovia, London, WC1E 7HB UK; 12 C/O National Cancer Research Institute, 407 St Johns Street, London, EC1V 4AD UK; 13 Department of Thoracic Medicine, University College London Hospital, 250 Euston Road, London, NW1 2PG UK; 14 Paul Strickland Scanner Centre, Mount Vernon Hospital, Middlesex, UK; 15 Department of Radiology, Royal Marsden NHS Foundation Trust, Fulham Rd London, London, SW3 6JJ UK; 16 Division of Cancer and Surgery, Faculty of Medicine, Hammersmith Campus, Imperial College London, London, W12 ONN UK

**Keywords:** Colorectal cancer, Lung cancer, Staging, Computed tomography, Whole body magnetic resonance imaging, Positron emission tomography, Patient experience

## Abstract

**Background and aims:**

Rapid and accurate cancer staging following diagnosis underpins patient management, in particular the identification of distant metastatic disease. Current staging guidelines recommend sequential deployment of various imaging platforms such as computerised tomography (CT) and positron emission tomography (PET) which can be time and resource intensive and onerous for patients. Recent studies demonstrate that whole body magnetic resonance Imaging (WB-MRI) may stage cancer efficiently in a single visit, with potentially greater accuracy than current staging investigations. The Streamline trials aim to evaluate whether WB-MRI increases per patient detection of metastases in non-small cell lung and colorectal cancer compared to standard staging pathways.

**Methods:**

The Streamline trials are multicentre, non-randomised, single-arm, prospective diagnostic accuracy studies with a novel design to capture patient management decisions during staging pathways. The two trials recruit adult patients with proven or highly suspected new diagnosis of primary colorectal (Streamline C) or non-small cell lung cancer (Streamline L) referred for staging. Patients undergo WB-MRI in addition to standard staging investigations. Strict blinding protocols are enforced for those interpreting the imaging. A first major treatment decision is made by the multi-disciplinary team prior to WB-MRI revelation based on standard staging investigations only, then based on the WB-MRI and any additional tests precipitated by WB-MRI, and finally based on all available test results. The reference standard is derived by a multidisciplinary consensus panel who assess 12 months of follow-up data to adjudicate on the TNM stage at diagnosis. Health psychology assessment of patients’ experiences of the cancer staging pathway will be undertaken via interviews and questionnaires. A cost (effectiveness) analysis of WB-MRI compared to standard staging pathways will be performed.

**Discussion:**

We describe a novel approach to radiologist and clinician blinding to ascertain the ‘true’ diagnostic accuracy of differing imaging pathways and discuss our approach to assessing the impact of WB-MRI on clinical decision making in real-time. The Streamline trials will compare WB-MRI and standard imaging pathways in the same patients, thereby informing the most accurate and efficient approach to staging.

**Trial registration:**

Streamline C ISRCTN43958015 (registered 25/7/2012). Streamline L ISRCTN50436483 (registered 31/7/2012).

## Background

Lung and colorectal cancer are the second and third most common malignancies in the UK, each accounting for 16% of all new cancer diagnoses; approximately 87,000 patients diagnosed per year [1]. Treatment decisions for both pivot on rapid and accurate tumour staging following diagnosis. In particular, detection of metastatic disease (i.e. disease which has disseminated beyond the primary tumour into distant organ sites such as bone, liver and brain) is vital for appropriate management.

In colorectal cancer, 50% of patients undergoing primary surgery with curative intent, relapse subsequently with metastatic disease, often within 12 months [2], in part reflecting sub-optimal staging following diagnosis. In lung cancer, over 20% of patients undergoing curative thoracotomy for presumed early stage disease relapse rapidly due to metastatic disease undetected by conventional staging tests – so called “futile thoracotomy” [3].

Cancer staging depends on high technology imaging platforms such as computerised tomography (CT), positron emission tomography (PET) and magnetic resonance imaging (MRI), together with standard scintigraphy, plain X- rays and ultrasound. Staging pathways are complex because these various modalities have differing diagnostic accuracies across different tumour types and body organs. The UK National Institute for Health and Care Excellence (NICE) guidelines outline an integrated but step-by-step sequential deployment of various imaging modalities before tumour staging is deemed complete and the first treatment decision is made [4]. It is not unusual for a patient with colorectal cancer to undergo CT chest, abdomen and pelvis, together with pelvic MRI in the case of rectal cancer, with additional tests such as ultrasound, MRI and PET-CT used increasingly in cases of known or suspected metastatic disease. Similarly, it is not unusual for a patient newly diagnosed with lung cancer to undergo standard chest and abdominal CT, whole body PET-CT, brain CT, and invasive mediastinal nodal sampling before the first definitive treatment decision. This stepwise multi-modality approach is both time and resource intensive, and onerous for patients at a very difficult time. Furthermore, patients receive significant doses of ionising radiation during the staging process that increase an individual’s risk of subsequent malignancy [5].

Recent technological advances allow whole body MRI scanning within one hour; sensitivity for metastatic disease is reported to be high [6–10]. MRI does not use ionising radiation and may be a safer, more efficient and accurate alternative to the standard multi-modality approach. There is little secondary research evidence concerning the accuracy of whole body MRI (WB-MRI) for cancer staging [11]. Meta- analysis is challenging because the WB-MRI primary literature comprises small single-centre cohort studies across a wide variety of cancers. These limited data do however indicate potential as an efficient and more accurate alternative for cancer staging. For example, NICE have recently recommended WB-MRI as first line in the initial staging of myeloma, due to higher accuracy compared to CT and standard skeletal survey [12]. The vast majority of available studies are however single site, using one MRI platform, with interpretation limited to one or two experienced radiologists. Generalisability is limited because most studies investigate single modality comparisons (e.g. WB-MRI vs. PET-CT) rather than comparing “real life” complex multi-modality staging pathways. Moreover, studies usually focus on diagnostic accuracy rather than therapeutic impact (i.e. the effect of testing on clinical decision making). Other limitations include frequent retrospective study designs, introducing bias.

This paper outlines the protocols of two multicentre studies comparing WB-MRI to standard NICE-approved diagnostic pathways for staging, (1) colorectal cancer –Streamline C (ISRCTN no: 43,958,015) and, (2) non-small cell lung cancer -Streamline L (ISCRTN no: 50,436,483). The studies are pragmatic and incorporate a variety of MRI platforms and interpreting radiologists, and assess the impact of WB-MRI on clinical decision making in real-time. In addition, the trials incorporate a health psychology assessment of patients’ experiences of WB-MRI, investigating potential impact on the physical and psychological burden of the cancer staging pathway, and predictors of patient preference. A detailed analysis of the cost and the cost-effectiveness of WB-MRI versus standard NICE-approved staging pathways will also be conducted. These trials are funded by the UK National Institute of Health Research (NIHR) Health Technology Assessment (HTA) programme, sponsored by University College London, and coordinated by the Cancer Research UK and UCL Cancer Trials Centre.

### Study objectives

#### Primary objective

To evaluate whether initial staging with whole body magnetic resonance Imaging (WB-MRI) increases per patient sensitivity for metastasis in colorectal and lung cancer compared to standard NICE-approved diagnostic pathways.

#### Secondary objectives

1. To determine how WB-MRI influences time to, and nature of, the first major treatment decision compared to standard investigations, and to determine whether early WB-MRI could reduce or replace standard investigations based on its diagnostic accuracy.
2. To assess the accuracy of WB-MRI and standard diagnostic pathways for local and distant cancer staging. WB-MRI will be evaluated both as an additional test to standard pathways, and as a replacement test based per organ and per metastasis analysis.
3. To determine the lifetime incremental cost and cost-effectiveness of staging using WB-MRI compared to standard diagnostic pathways.
4. To evaluate patients’ experiences of staging using WB-MRI and to determine the priorities they place on differing attributes offered by competing staging pathways, including impact of reducing time to first treatment.
5. To determine the inter-observer variability of WB-MRI diagnosis of metastatic disease by different radiologists and their confidence in diagnosis.
6. To evaluate the diagnostic accuracy of WB-MRI protocols limited to diffusion and T1 weighted imaging only, and to assess the incremental benefit on diagnostic accuracy of intravenous gadolinium contrast enhancement.

## Methods

### General

Streamline C and Streamline L are parallel multi-centre prospective cohort studies comparing the staging accuracy of WB-MRI with standard pathways for newly diagnosed colorectal (Streamline C) and non-small cell lung cancer (Streamline L) conducted at 25 English National Health Service (NHS) hospitals. Data investigating the therapeutic impact of WB-MRI on patient management compared to standard pathways will be collated. The trial design compares two different staging strategies in the same patients ensuring comparable data are collected for both pathways while simultaneously increasing trial power to meet endpoints by using paired data.

Both qualitative and quantitative assessments will be employed to determine the psychological burden and acceptability of WB-MRI versus standard pathways, and to identify those attributes that influence patient preference most strongly. Health-related quality of life data is being collected, which will be used to inform cost-effectiveness modelling.

### Inclusions/exclusion criteria

Inclusion and exclusion criteria are outlined below. A series of imaging hubs will perform the WB-MRI according to the trial protocol (see below). Patients will be recruited from local hospitals (recruitment sites) and referred to the closest imaging hub for the WB-MRI. Some hospitals will act as both recruitment sites and imaging hubs.

In the colorectal cancer trial (Streamline C), eligible patients are aged 18 or older, able to give written informed consent, with histologically proven or strongly suspected colorectal cancer referred for staging (the latter defined as the presence of a mass highly suspicious for colorectal cancer on endoscopy, barium enema, CT colonography or other imaging which triggers staging investigations).

Patients with polyp cancer will be excluded because metastatic disease in these patients is exceedingly rare.

In the lung cancer trial (Streamline L), eligible patients are aged 18 or older, able to give written informed consent, with histologically proven or clinically diagnosed primary non-small cell lung cancer (NSCLC) with potentially radically treatable disease. Clinically diagnosed NSCLC cancer is defined as radiological diagnosis of lung cancer on chest CT with sufficient confidence to trigger staging investigations. Potentially radically treatable disease is defined as stage IIIb or less on diagnostic CT (i.e. T1–4, N0–2, M0). Patients should have a performance status 0 to 2 inclusive (and fit to undergo radical treatment if indicated). Patients with unequivocal metastatic or N3 disease on diagnostic CT chest and abdomen (including M1a disease; malignant pleural effusion) will be excluded as will those in whom further staging work up is not indicated due to poor performance status or patient choice. Those with histology other than non-small cell lung cancer will also be excluded given the differing biological behaviour of small cell lung cancer.

For both Streamline C and Streamline L, patients will be excluded if they have any psychiatric or other disorder likely to impact on informed consent, or evidence of severe or uncontrolled systemic disease, which make it undesirable for the patient to participate in the trial. Pregnant patients or others with contraindications to MRI (e.g. cardiac pacemaker, severe claustrophobia, inability to lie flat) are also excluded.

### Ethical arrangements and consent

The Streamline trials were approved by the London – Camden and King’s Cross Research Ethics Committee on 3rd October 2012 and are being conducted in accordance with the principles of ICH guidelines on good clinical practice in clinical trials and the Research Governance Framework for Health and Social Care (England). All patients give written informed consent prior to inclusion in the trials.

### Diagnostic interventions

#### Whole body MRI (WB-MRI)

The choice of MRI platform (i.e. manufacturer and Tesla (T) strength) will be decided by the local hub radiologist according to scanner availability and their usual clinical practice; either 1.5 T or 3 T platforms can be used. WB-MRI will be performed no later than 3 weeks after the final standard staging investigation. A minimum sequence dataset will be acquired including standard T1, T2 axial sequences supplemented by diffusion weighted (minimum 2 b-values, 50 and 900 s/mm^2^) and contrast enhanced T1 images through the liver, lungs and brain.

#### Standard imaging

Recruited patients will undergo all standard staging investigations employed at their hospital as per local protocols and requirements for usual care. The nature and date of these standard investigations (e.g. CT scan, PET-CT, organ specific MRI, biopsy etc.) will be recorded, along with the presence and location of any metastatic disease based on the radiological report, for later comparison.

### Blinding of WB-MRI reporters

WB-MRI will be reported by radiologists blinded to the results of standard imaging tests and other clinical information (other than the cancer diagnosis and site of the primary tumour). A novel approach employing a secure central imaging server (3Dnet™) provided by Biotronics3D (London, UK) has been employed to ensure the required level of blinding. Specifically, WB-MRI datasets will be anonymised by radiographer/technologists before upload from each hub to the secure central imaging server (3Dnet™). This solution enables rapid and simple upload of complex imaging datasets via a standard internet connection. Unanonymised WB-MRI images will only be released to the hospital picture and archiving system (PACS) after their revelation at the multi-disciplinary team (MDT) meeting (see below) so those reporting standard staging investigations are blinded to the WB-MRI images and findings.

### Interpretation and reporting

WB-MRI will be interpreted by designated trial radiologists who have prior experience in interpretation, defined as at least 20 validated WB-MRI cases in patients with lung or colon cancer. Radiologists with experience of less than 100 WB-MRI datasets will in addition undergo a period of “buddy” reporting with a more experienced radiologist (>100 WB-MRI datasets), and will only report alone once deemed competent by the more experienced radiologist. Interpretation may be performed using the 3Dnet™ viewing software or local workstations after download of the WB-MRI images, according to radiologist preference. A sequential viewing paradigm with ordered reading of selected sequences will used to examine the contribution of individual MRI sequences to radiologist diagnostic confidence and accuracy.

A free text clinical report will be produced based on all available sequences containing information relating to the local tumour (T), its nodal (N) stage, together with the presence, location, number and size of any metastatic deposits (M), as well as clinically important incidental findings, for example aortic aneurysm. The radiologist may recommend additional tests for equivocal findings as per their routine clinical practice.

### MDT review and influence of imaging on patient management

As per usual clinical practice, the MDT meeting will review all standard staging imaging initially blinded to the WB-MRI findings. MDT meetings will occur at least fortnightly, and usually weekly in a dedicated meeting room and as a minimum will include a radiologist, oncologist, histopathologist and respiratory physician and/or surgeon (Streamline L) or colorectal surgeon (Streamline C). It is anticipated that most MDT meetings will include more than one representative of these specialities. The MDT will have access to the full clinical record and histopathological data via the patient notes or electronic patient record, together with all standard imaging and reports via PACS. Based on this alone, the MDT will document the first major treatment decision (e.g. referral for surgical excision of the primary tumour, instigation of definitive treatment using chemotherapy, palliative/supportive care etc), as per usual care, along with the TMN stage.

The WB-MRI report and images will then be revealed to the MDT members by the MDT coordinator or designated representative via 3Dnet™ using an internet enabled PC (or via written paper copies in cases of IT failure). MDT members will evaluate the WB-MRI report and document any additional tests they deem the WB-MRI result generates (if any). If these additional tests have already been performed as part of the standard investigations, the MDT will then document the theoretical first major treatment decision based on the WB-MRI and results of the tests it would have generated. Finally, the MDT will then document their definitive first major treatment decision based on all available information (including the WB-MRI).

If the additional tests generated by the WB-MRI result have not already been performed as part of standard investigations and are deemed necessary by the MDT, these will be performed and the patients re discussed at the next scheduled MDT meeting when the process described above will be followed.

Occasionally a patient may commence definitive treatment before the date of a scheduled MDT such that the WB-MRI (and/or generated additional tests) will not yet have been revealed to the MDT. In this scenario, an ad hoc MDT will be convened consisting of all specialities relevant to the patient’s clinical care, and the MDT process described above will be followed.

### Early release of WB-MRI findings

The WB-MRI images and reports may be released early by reporting radiologists (ie before controlled revelation at the MDT meeting) if a diagnosis is made that potentially requires urgent intervention (specifically impending spinal cord compression, deep vein thrombosis or pulmonary embolism, or brain metastasis with significant mass effect requiring immediate treatment), or on request of the patient’s lead clinician for the patient’s clinical care in an emergency clinical situation. If early release precludes unbiased MDT assessment of the standard staging investigations, then the patient will be replaced.

### Time for staging

The total time taken to fully stage the patient using standard staging pathways will be calculated from the request date of the first staging investigation to the date of the MDT meeting’s first major treatment decision based on the standard imaging pathway. For Streamline L, the start of the staging process will be defined as the request date of the first staging investigation following a proven or highly suspected diagnosis of lung cancer (for example the date of requested PET/CT after diagnostic chest CT). For Streamline C, the start of the staging process will be defined as the request date of the first staging investigation following a proven or highly suspected diagnosis of colorectal cancer (for example the date of request for CT chest, abdomen and pelvis after colonoscopic diagnosis of likely malignant tumour). Because revelation of WB-MRI is deferred until after standard staging is complete, precise measurement of time to full staging using WB-MRI will not be possible. The theoretical time to complete staging using WB-MRI will be thus modelled taking into consideration the time from recruitment date to the date of the WB-MRI, plus the number and type of additional staging investigations generated by WB-MRI.

### Patient follow-up

Patients will be followed for a period of 12 months from the date of consent. Patients are asked to complete a resource use diary every 3 months (recording all hospital and GP visits, community care, medications and investigations) and complete a validated general health questionnaire (EQ-5D) (http://www.euroqol.org/) every three months for subsequent health economic analysis.

### Health psychology assessment

Health psychology assessment is essential for comprehensive evaluation of cancer staging pathways. A subset of up to 25 patients for each cancer type will be interviewed to determine their experience of WB-MRI and standard staging tests, and also cancer staging pathways in general. Interviews will assess which aspects of testing cause patients most physical or psychological stress (e.g. number of tests/ hospital visits, test attributes such as claustrophobia, need to lie still, etc.), and will elicit any factors that patients feel would have made staging easier. These qualitative data will inform the design of a patient experience questionnaire and, thereafter, a discrete choice experiment (DCE). It is planned to administer the questionnaire until 50 patient responses are collated both before and after the WB-MRI, by which point we anticipate saturation will have occurred. The DCE will be targeted at 50 patients for each cancer type and aims to elicit preferences for staging pathways by estimating the relative importance of different attributes, and the trade-offs between them. WB-MRI and standard staging pathways differ in associated attributes, not only related to physical experience but also rate of adverse events, time to diagnosis, and overall accuracy etc. The most important attributes will be identified in the interview and questionnaire studies and appropriate levels assigned to each based on accumulating data from the trials, together with appropriate literature review. A DCE questionnaire will be developed whereby patients state their preference between two choices, with each choice containing different levels of the identified attributes. Comparisons will be repeated a number of times depending on the number of attributes and levels identified.

Sample size for the discrete choice experiment should be greater than (500*c)/(t*a) where t = the number of sets of choices, a = the number of scenarios to choose between in each choice, and c = the largest number of levels for any one attribute. Assuming each patient will undertake 15 sets of choices, there are two scenarios in each choice, and the largest number of levels for any one attribute is 3, the required sample size is (500*3)/(15*2) = 50 per cancer type.

Further questionnaires including patient satisfaction, the positive and negative affect schedule (PANAS) [13] and the General Health Questionnaire (GHQ-12) [14] will be administered during and after staging pathways, and at three and six months later to examine how patient test preferences may change as their treatment trajectory progresses.

### Clinical follow-up

MDT records and hospital data repositories will be used over the 12-month follow-up period to collate primary histological stage (where surgery performed), the results of any biopsy procedure, and details and findings of follow-up imaging investigations (in particular the presence or absence of metastasis). The date and cause of patient death and post mortem findings (if performed) will also be recorded.

### Final reference standard for tumour stage

Multi-disciplinary consensus panel review is standard methodology for diagnostic test accuracy studies where an independent reference standard does not exist, or is impossible because of incorporation bias. Consensus panels will convene regularly to derive the reference standard for tumour stage at diagnosis for recruited patients completing 12-months of follow-up. The panels will consider all available clinical information including the results of all original standard staging investigations, WB-MRI, histopathology (surgical resection and biopsies), follow up imaging, post-mortem reports (where available) and MDT meeting records. Each panel consists of at least an oncologist, and/or a colorectal surgeon, and a minimum of two radiologists, one external to the recruitment site and one internal. At least one radiologist must have specific expertise in WB-MRI and in PET-CT. It is not necessary for this radiologist be interpreting WB-MRI as part of the trials. The panel will have access to a histopathologist if required. The purpose of the panel is to adjudicate on the TNM stage of the cancer at diagnosis, including the organ specific sites and burden of metastatic spread against which the accuracy of WB-MRI and standard staging pathways are compared.

### Cost effectiveness

Resource use data for the main drivers of hospital costs will be collected from patient records, notably number of outpatient visits, inpatient hospital stays, interventional procedures including surgery, repeat imaging investigations and treatment with chemotherapy or radiotherapy. In addition, patients are requested to complete resource use diaries at baseline, and at 3, 6, and 9 months. The diaries will be used to collect data on primary and community care contacts during the 1-year period of follow up period. Additionally, health-related quality of life score, measured according to the EQ-5D, will be measured at baseline and at 3, 6, 9 and 12 months for all surviving patients.

### Outcome measures

#### Primary outcome measure

Per patient sensitivity for metastasis detection by WB-MRI compared to standard staging pathways in newly diagnosed colorectal and non-small cell lung cancer.

#### Secondary outcome measures

1. The time and test number taken to reach, and the nature of, the first major treatment decision based on WB-MRI compared to standard staging pathways
2. Diagnostic accuracy of WB-MRI and conventional staging pathways for local tumour staging and detection of metastasis. WB-MRI will be evaluated both as an additional test to standard pathways per patient, and as a replacement test based on per organ and per metastasis analysis.
3. The lifetime incremental cost and cost-effectiveness of staging using WB-MRI compared to standard diagnostic pathways
4. Comparative patient experiences of staging using WB-MRI and standard investigations, and the average relative importance weighting of attributes ascribed to standard versus WB-MRI staging pathways.
5. Inter-observer variability in WB-MRI accuracy and effect of radiologist diagnostic confidence on staging accuracy.
6. Diagnostic accuracy of WB-MRI limited to T1 and diffusion weighted sequences compared to full WB-MRI protocols.

### Sample size

For both trials, study power is based on a difference in sensitivity for metastases detection, with the WB-MRI pathway replacing the standard staging pathway. Both trials are powered at 80% for type II error and 5% for type 1 error. The sample size was calculated assuming WB-MRI is more sensitive than standard staging pathways, and accounting for the expected prevalence of metastasis in study cohorts [15].

For Streamline C, assuming WB-MRI has 10% greater sensitivity for metastasis than standard pathways (85% vs. 75%), a 40% prevalence of metastasis, 73% concordance between WB-MRI and standard staging in patients deemed to have metastatic disease by the consensus panel, and assuming 10% withdrawal rate, we need to recruit 322 patients, giving 290 evaluable patients (i.e. patients with a cancer diagnosis and completing WB-MRI with sufficient follow-up to assign a consensus reference stage).

The initial recruitment target for Streamline L was 250 patients including 200 evaluable patients, based on 24% greater WB-MRI sensitivity for metastasis (79% WB-MRI, 55% standard staging), 25% prevalence of metastasis and 53% concordance between WB-MRI and standard staging in patients deemed to have metastatic disease by the consensus panel. A withdrawal rate of 20% was assumed.

During trial recruitment, and based on advice from the independent data monitoring committee, recruitment targets were increased to 360 (giving 290 evaluable patients) for Streamline C and 353 (giving 200 evaluable patients) for Streamline L because of higher than expected withdrawal rates of 19% and 43% respectively. The higher withdrawal rate was predominantly due to either a final non-cancer diagnosis, or failure to undergo or complete the WB-MRI scan.

### Analysis

A detailed statistical analysis plan will be produced and finalised prior to data lock and transfer to trial statistician. Analysis will consider all patients in the study using multiple imputation to account for missing data with a sensitivity analysis based on complete case analysis.

Analysis for the primary outcome will use multivariate logistic regression of paired binary outcomes for comparison of sensitivity and specificity of WB-MRI and standard investigations within patients. 95% confidence intervals will be calculated and *p*-values <0.05 will be considered statistically significant. A similar approach will be used for secondary outcomes.

There will be no adjustment of *p*-values for secondary outcomes for multiple testing. STATA (Statacorp LP, Texas, USA) statistical software will be used.

Health economic analysis will be performed by calculating (for each individual patient) the lifetime costs and the quality-adjusted life-years (QALYs) experienced. Costs extrapolated beyond the time horizon of one-year follow-up will be deduced by development of a de novo cost-effectiveness model for the disease pathways, which will be populated via available evidence observed from the trials. Individual patients will then be grouped according to the specific disease path and the accuracy of the staging result. We will calculate mean costs and QALYs for each group and for WB-MRI versus standard staging algorithms.

Analysis of health psychology interviews will unearth the potential options for the health psychology questionnaire and the discrete choice experiment. Comparative patient experience between WB-MRI and standard staging investigations, identification of important staging pathway attributes, comparative anxiety, expectations and attribute importance before and following the staging process will be analysed using appropriate comparative and regression statistics.

## Discussion

At the time of writing, the Streamline trials are the largest prospective multicentre trials to directly compare WB-MRI with standard imaging pathways for cancer staging. The trials will be particularly informative because they compare comprehensive staging pathways rather than binary comparison of single imaging test platforms. Pathway comparisons are made at relevant points in the patient’s trajectory and recruits are representative of patients undergoing WB-MRI in clinical practice should it be introduced into the NHS. These trials will therefore provide valuable information to guide implementation of high technology imaging platforms for cancer staging.

The trial design includes two important features: methods to ensure blinding of those interpreting trial imaging; and evaluation of the impact on therapeutic management decisions made real time in the context of the MDT meeting. The latter in particular is unusual in imaging trials of this size. Furthermore, the trials include a discrete choice experiment for patient preferences, again an infrequent component of imaging technology trials. We employ a reference standard based on consensus panel meetings, to allow assessment of potential incorporation bias, as discussed below.

### Blinding of trial imaging

Ascertaining the true standalone diagnostic accuracy of a novel imaging test demands removal of certain external influences on radiological decision making. Interpretation of WB-MRI is likely to be influenced by knowledge of other competing staging tests, to which the reporting radiologists will therefore be blinded. However, to mimic normal clinical practice, those interpreting WB-MRI will not be blinded to the cancer diagnosis nor the site of the primary tumour [16]. The WB-MRI images and reports must not be immediately available to those radiologists reporting the standard staging investigations, nor those involved in direct patient care before the patient has been fully staged using standard investigations such that the first major treatment decision can be made uninfluenced by the WB-MRI findings. This approach ensures the standard staging pathway are captured “intact” before revelation of the WB-MRI results.

The PACS is the usual repository for imaging studies in the NHS and accessible to many hospital staff. To send un-anonymised WB-MRI images immediately to the local PACS for reporting would potentially unmask blinding. Instead, anonymised image data will be uploaded at each imaging hub to a secure central imaging server (3Dnet™) provided by Biotronics3D. A computer based internet gateway will be installed in each imaging hub to facilitate automated transfer of WB-MRI from the scanner/workstation to 3Dnet™ for reporting and storage, and thereafter automatically back to PACS at the appropriate time point.

The 3Dnet™ server will also facilitate simple and timely release of the WB-MRI report and images to the MDT meeting without the need for CD transfer.

### Comparison of real-time clinical impact of diagnostic pathways

The trials are designed to compare the impact of the WB-MRI staging pathway on patient management with that based on standard imaging in the context of an MDT. Impact is captured in real-time, contemporaneous within the patients’ actual clinical care pathway determined at the MDT. This design also facilitates health economic analysis as it provides prospective data on patient management decisions made as a direct result of the staging test results.

Specifically, WB-MRI imaging and reports are only revealed once the MDT has committed itself to a full staging and treatment decision based on standard investigations, but prior to any treatment being instigated. After WB-MRI has been revealed the MDT will state whether, based on WB-MRI alone, the patient had been adequately staged or whether additional tests are required to clarify potential pathology indicated by WB-MRI findings.

The final treatment plan for each patient will be based on all available information, incorporating both standard imaging pathways and WB-MRI. This design is necessary because it would be unethical to not act on potentially important WB-MRI findings. It will therefore be possible to capture the following:

- Treatment decision based on standard investigations alone (and the number, timing nature and findings of these investigations).
- Treatment decision based on WB-MRI and any additional staging investigations generated by the WB-MRI (and the number, nature and findings of additional tests generated).
- Final treatment decision incorporating information from all available tests.

In addition, record will be made of any complications attributable to staging pathways, notably contrast reactions, biopsy complications (infection, bleeding or hospital admission), and delays in commencing definitive treatment.

### Consensus panel as reference standard and assessment of incorporation bias

There is no single reference standard test for the initial staging of lung and colorectal cancer. In such cases it is acceptable design to convene a consensus to judge the presence or absence of the target condition based on multiple sources of information. In Streamline, panels will consist of a variety of personnel from different disciplines allowing all imaging modalities and clinical data to be discussed with equal emphasis informed by strict guidance for the classification of new lesions identified during the follow-up period, or indeterminate lesions.

To standardise panel decisions, a member of the central trial team will attend each individual consensus meeting to ensure similar criteria are used across centres to define disease extent. The comparative findings of all staging and follow up investigations (including all imaging) will be documented to allow estimation of any potential incorporation bias in the final consensus panel decision.

### Discrete choice experiment

Discrete choice experiments (DCE) will be designed to elicit patient preferences for the different attributes of WB-MRI and conventional staging pathways (such as time to diagnosis, exposure to radiation, overall accuracy etc.) [17]. DCE designs elicits preferences from patients regarding competing attributes. For example, what is most important; a single visit to the hospital or a more accurate testing pathway but which requires several visits? Those attributes most important to patients will be identified via interviews and questionnaires. Appropriate levels will be assigned to each based on accumulating data from the trial and appropriate literature review. This information will populate the DCE questionnaire which will ask patients to state their preference between two competing choices, with each choice containing different levels of the identified attributes. Comparisons will be repeated a number of times depending on the number of attributes and levels identified in order to identify the “tipping” point, i.e. that point at which the patient switches to favouring one attribute over another*.*

## Conclusion

The Streamline colorectal and lung cancer trials are multi-centre prospective cohort studies comparing the diagnostic accuracy of WB-MRI with standard staging tests for initial tumour staging. The trials include important design features such as real time capture of patient management decisions based on staging test results, allowing impact comparisons for therapeutic decision making. Novel approaches for blinding of image interpretation are employed and in depth patient experience information will be collected to analyse the acceptability of the two separate pathways.

### Trial status

The Streamline trials have completed recruitment. Streamline C opened on 8th March 2013 and closed to recruitment on 19th August 2016, recruiting 370 patients. Streamline L opened on 7th February 2013 and closed to recruitment on 5th September 2016 recruiting 353 patients. The analysis plan described in this protocol will be implemented in due course after data retention, follow up and cleaning.

## Abbreviations

CT: Computerised tomography
HTA: Health Technology Assessment
M: metastatic deposits
MDT: multidisciplinary team
MRI: magnetic resonance imaging
N: nodal stage
NICE: The UK National Institute for Health and Care Excellence
NIHR: National Institute of Health Research
PACS: picture archive and communication system
PET: Positron Emission Tomography
REC: Research Ethics Committee
T: local tumour stage
T: Tesla
WB-MRI: Whole Body Magnetic Resonance Imaging
